# Research highlights, *The Plant Genome*, Volume 15, Issue 1

**DOI:** 10.1002/tpg2.20203

**Published:** 2022-03-20

## 1 GEPSDB: THE GENE EXPRESSION DATABASE OF POPLAR UNDER STRESS

As a model tree species, poplar has important economic and ecological value. Here, Liu et al. (https://doi.org/10.1002/tpg2.20163) constructed the GEPSdb (Gene Expression Database of Poplar under Stress (http://gepsdb.ahau‐edu.cn/), which is an integrated database of poplar gene expression profiles derived from RNA‐seq and microarray library data. The GEPSdb is a resource of gene expression profiles under abiotic and biotic stress. In this study, 41,335 genes were detected to be expressed employing both microarray and RNA‐seq datasets. Comprehensive information on individual genes was also provided in GEPSdb.

## 2 BANANA STUDY REVEALS HIDDEN GENETIC DIVERSITY

Commercially grown bananas have very little diversity, making them susceptible to disease, which can devastate entire populations. The identification of novel disease resistance genes is a priority for commercial banana breeding. Rijzaani et al. (https://doi.org/10.1002/tpg2.20100) examine the genomes of diverse fruit‐producing *Musa* banana from Asia and *Ensete* banana from Africa that are grown for their starchy corm. The gene content clearly distinguishes the two groups, with several hundred genes unique to each group. These findings can be used to understand the diversity of disease resistance and fruit quality traits in banana, supporting breeding programs in these crops.

## 3 GENE SELECTION DURING SOYBEAN BREEDING

Bayer et al. (https://doi.org/10.1002/tpg2.20109) examine the impact of soybean domestication and breeding through the analysis of >1,000 sequenced lines from the U.S. soybean collection. Through the assembly of a pangenome, they identify variable genes and alleles that have significantly changed in frequency during breeding, reflecting selection for adaptation and improved traits. They show that the genomes of modern U.S. cultivars contain fewer genes than older cultivars, an unexpected result of selection. Changes in gene and allele frequency suggest new approaches for further improvement in this important crop.

## 4 MACHINE LEARNING FOR FAST PANGENOMICS

Genes are either present in all members of a population (core) or absent in at least a single individual (dispensable). Parsing genes into these categories requires expensive surveys of many individuals. Yocca and Edger (https://doi.org/10.1002/tpg2.20135) report machine‐learning models are able to identify genes as core or dispensable using only a single reference genome. Perhaps in the future, this method will assist genomic investigations in underdeveloped and underfunded systems.

## 5 RARE OR NOVEL *RHG1* DISCOVERED IN WILD SOYBEAN

Soybean cyst nematode (SCN) is the most yield‐damaging disease of soybean, and *Rhg1* encodes SCN resistance. From available SoySNP50K single‐nucleotide polymorphism data on >1,000 wild soybean accessions, Grunwald et al. (https://doi.org/10.1002/tpg2.20152) used presence of an SCN resistance‐associated gene not linked to *Rhg1* to identify a small set of candidate accessions and then examined their *Rhg1* loci in detail. They discovered and characterized a novel variant of the *Rhg1* α‐SNAP SCN resistance protein that is rare in (not present in most accessions of) the wild soybean relative *Glycine soja*. They also demonstrated that the unusual *Rhg1* locus structure, with copy number variation of a four‐gene block, arose prior to the domestication of soybean. *Glycine soja* accessions with potentially valuable SCN resistance were identified.

## 6 COMMON BEAN RESPONSE TO *MELOIDOGYNE INCOGNITA*

The common bean crop is severely affected by root‐knot nematode attacks, leading to huge yield loss in developing countries. Even though crop resistance is an outstanding strategy for controlling nematode damage, the genetic basis of the common bean response is poorly understood. Giordani et al. (https://doi.org/10.1002/tpg2.20161) identified eight independent genomic regions associated with egg‐mass production and root‐galling index, suggesting that complex mechanisms are involved in suppressing nematode infection. Also, resistance gene analogs and genes related to hypersensitive responses were found within the associated regions, opening new opportunities for dissecting the molecular mechanisms underpinning the resistance and contributing to common bean breeding.

## 7 DYNAMICS OF SINGLE‐NUCLEOTIDE MOSAICISM IN CITRUS

It is currently accepted that plants continuously accumulate somatic mutations. Most citrus varieties, for instance, are invariably derived from spontaneous mutations. Perez‐Roman et al. (https://doi.org/10.1002/tpg2.20162) have performed whole‐genome sequencing on different leaf flushes of a single clementine tree to elucidate the dynamics of mosaicism in citrus. The average estimate of the mutation rate in this tree was 4.4 × 10^−10^ bp^−1^ yr^−1^. It was also determined that an ideal tree of similar age carried a total of 1,500–5,000 variants, while each axillary meristem produced, on average, one somatic mutation. The data indicated that somatic mutations spread following an iterative pattern determined by the sympodial model of branching of citrus, and that the mutations consequently accumulated in sectorized areas of the tree in a nested hierarchy. A citrus tree, therefore, is a mosaic genetically composed of different genomes distributed by sectors.

## 8 SELECTION SIGNATURES IN CIMMYT YIELD TRIALS

Two historical sets of wheat lines were genotyped to explore trends of genetic diversity and selection footprints associated with continuous crop improvement and environmental adaptation. Wheat lines displayed CIMMYT germplasm globally distributed since 1979 and adapted to two different mega‐environments (irrigated, high‐production and low‐rainfall environments). Mondaini et al. (https://doi.org/10.1002/tpg2.20165) found high levels of admixture, indicating that the entire genetic diversity present in the germplasm pool is harnessed to target core traits to individual mega‐environments. Genome‐wide scans revealed that 9.8 and 2.0% of the SNP markers could be associated to selection signatures over time and to environmental adaptation, respectively. Several known genes and previously identified haplotypes associated with grain yield in more recent CIMMYT elite germplasm did fall into genomic regions with directional selection.

## 9 MTP IDENTIFICATION IN CUCURBITACEAE SPECIES

Metal‐tolerance proteins (MTPs) are divalent cation transporters and play fundamental roles in plant metal tolerance and ion homeostasis, but a systematic investigation of MTPs in Cucurbitaceae is still lacking. Jiang et al. (https://doi.org/10.1002/tpg2.20167) identified 142 MTPs from 11 released genomes of eight Cucurbitaceae species and analyzed their phylogenetic relationships, gene structures, chromosome distributions, conserved domains, and motifs. RNA‐seq data analysis revealed the diverse expression patterns of MTP genes in five Cucurbitaceae species across different tissues and developmental stages. The qPCR analysis demonstrated the expression profiles of all of the cucumber MTP genes in response to different heavy metal stressors (Cd, Mn, Zn, and Cu), and the expression levels of all CsMTP members were induced by at least one metal ion. This study provides essential insights into Cucurbitaceae MTP genes and lays the foundation for more in‐depth experimental exploration of the functions of these MTP genes.

## 10 RICE YIELD MAINTENANCE UNDER DROUGHT

Rice production around the world is increasingly being undermined by drought. As the global population steadily increases toward 10 billion, food security, especially in drought‐prone areas across the poorest regions of the world, will be a continual challenge. Sanchez et al. (https://doi.org/10.1002/tpg2.20168) report that in rice, expedient transition to reproductive growth while under prolonged drought leads to lower penalty to grain yield. By QTL (*qDTY12.1*) introgression breeding coupled with transcriptomics and genetic network modeling, they discovered that the *DECUSSATE* gene functions as core of regulatory mechanism that mediates growth adjustment toward early transition from vegetative to reproductive, causing significant reduction in yield losses under persistent drought. This adjustment represents an escape mechanism that conserves cellular resources for reproduction before they are severely exhausted because of stress effects. Proper functioning of this network is dependent on the synergy between *DECUSSATE* in *qDTY12.1* and a subset of flowering and seed development genes across the genetic background that are all controlled by the hormone cytokinin.

## 11 INTRAPLANT GENETIC DIVERSITY IN CANNABIS

Cannabis growers have been reporting a decay in quality from clones taken from aging mother plants but are uncertain on the underlying mechanism responsible. The observations made usually include a decline in plant vigor and cannabinoid levels. Adamek et al. (https://doi.org/10.1002/tpg2.20169) discovered varying amounts of somatic mutations within a singular mother plant, with the most detected at the uppermost sample. Deep whole‐genome sequencing (>50×) revealed millions of sequence variants based on a reference genome, and bioinformatics tools allowed the analysis of mutations at critical cannabinoid–terpene biosynthesis pathways. Findings indicated that genetic diversity exists within a single mother plant and suggests that the upper ends are more genetically distinct from the original plant than lower regions. As a result, the best practice for renewing mother plants would be to take a clone from a lower section to minimize the variation.

## 12 DEVELOPMENT OF CLIMATE‐RESILIENT RICE

Rice, an important and widely consumed cereal crop, is particularly vulnerable to biotic and abiotic stressors. Occurrence of multiple abiotic and biotic stresses has necessitated the development of climate‐smart rice by combining trait‐specific QTL and genes in high‐yielding cultivars to confer a wider range of tolerance or resistance. Enhanced capability of climate‐smart cultivars would enable the crop to thrive under adverse environmental conditions. Singh et al. (https://doi.org/10.1002/tpg2.20170) combined multiple genes and QTL for drought, bacterial leaf blight (BLB), and blast resistance genes in rice cultivar Lalat using marker‐assisted forward breeding (MAFB). The developed introgression lines (ILs) were phenotypically screened under reproductive stage drought stress and screened for BLB and blast. Introgression lines having targeted gene and QTL showed strong resistance or tolerance against the phenotyped traits against drought, BLB, and blast. Because of stringent phenotypic selection, the grain quality attribute (hulling, milling, head rice recovery, chalkiness, alkali spreading value, and amylose content) of ILs were found similar to elite cultivar type. According to the study, MAFB combined with simultaneous crossing proved to be an effective technique for rapidly combining multiple stresses in rice. The study showed that MAFB together with simultaneous crossing would be an effective strategy to rapidly combine multiple stresses in rice. The ILs developed in this study could contribute to the sustainability of yield in changing environments.

## 13 BIN‐MAPPING‐BASED QTL ANALYSIS UNCOVER MULTIPLE GRAIN‐SHAPE‐HETEROSIS‐RELATED LOCI IN RICE

Sixteen grain‐shape‐related QTLs and 30 heterosis‐related QTLs for grain‐shape‐related traits were detected. The mapped QTLs not only have overlap with known genes but also have new loci, such as *LOC_Os06g14260*, which encoded a putative lectin‐like receptor, and *LOC_Os04g51950*, which encoded a hypothetical serine–threonine protein kinase HTI. The new grain‐shape‐related genes mined by Deng et al. (https://doi.org/10.1002/tpg2.20171) provide new genetic loci for rice grain shape molecular design and breeding.

## 14 SEED COAT COLOR GENETICS BY MACHINE LEARNING

The L*a*b* color values measured at a pixel level are useful in evaluating bean seed coat color. However, the corona and hilum ring on bean seeds are noise in image analysis. Sadohara et. al. (https://doi.org/10.1002/tpg2.20173) measured the L*a*b* values of a yellow bean diversity panel by using machine learning, separating the seed coat from nontargets. The environmental influence on yellow seed coat color was independent of postharvest darkening type. Postharvest darkening in yellow bean was associated with the ground factor *P* gene. These results will help breeders in developing bean cultivars with desired seed morphological traits.

## 15 GENETIC DISSECTION OF LIGNOCELLULOSIC COMPOUNDS AND FERMENTABLE SUGARS IN RICE STRAW

Lignocellulosic compounds are the main components of secondary plant cell walls with importance for stem resistance and industrial processing. Also, the fermentable sugars are important for different industrial applications. Genetic dissection of cellulose, lignin, and fermentable sugar contents in rice straw was conducted using genome‐wide association study (GWAS). Panahabadi et al. (https://doi.org/10.1002/tpg2.20174) reported seven, three, and three genomic regions (QTL) significantly associated to cellulose, lignin, and fermentable sugar contents, respectively. Several candidate genes in the cellulose‐associated QTL intervals were identified with being enzymes mainly involved in cell wall metabolism including GH16, peroxidase, GT6, GT8, and CSLD2. Villin protein, OsWAK1/50/52/53, and GH16 were more important genes found in lignin‐associated QTL regions, and finally, UTP‐glucose‐1‐phosphate uridylyltransferase, BRASSINOSTEROID INSENSITIVE 1, and receptor‐like protein kinase 5 were found in the vicinity of QTL associated to fermentable sugar content.

## 16 *ELEUSINE* GENETIC AND TRAIT MAPPING

Finger millet [*Eleusine coracana* (L.) Gaertn. subsp. *coracana*] is a crucial cereal crop in eastern Africa and southern Asia but lacks genomic resources and modern breeding programs. Pendergast et al. (https://doi.org/10.1002/tpg2.20175) used genotype‐by‐sequencing (GBS) and an iterative genetic mapping pipeline on a F_2:3_ population to create a high‐density genetic map for finger millet, which allowed comparisons across rice, foxtail millet, and sorghum genome organization. Quantitative trait loci (QTL) analyses using trait data from a Kenyan field trial uncovered significant QTL for flowering date, plant height, panicle number, and blast incidence and severity. Most notably, seven likely disease‐resistance‐related LEUCINE‐RICH REPEAT‐CONTAINING PROTEIN genes were discovered under blast incidence and severity QTL. The genetic map and candidate genes may aid future breeding programs and help to better understand genetic mechanisms of blast resistance.

## 17 UNLOCKING SORGHUM'S SECRETS FOR DROUGHT ADAPTATION

Sorghum is known as one of the world's toughest crops, making it a critical staple for smallholder farmers in Africa who face severe and unpredictable droughts. Unfortunately, our limited understanding of sorghum's drought‐tolerance mechanisms has slowed efforts by African breeders to develop more climate‐resilient varieties. To address this knowledge gap, Faye et al. (https://doi.org/10.1002/tpg2.20176) characterized the drought response of hundreds of African sorghum landraces and discovered genomic variants associated with high yields under a range of drought scenarios. Notably, the findings suggest that some ‘stay‐green’ drought‐tolerance alleles, which had been studied in U.S. breeding lines, may already have been selected in West African landraces during the historical process of crop diffusion across Africa. These genetic discoveries provide a path for African breeders to leverage traditional varieties and marker‐assisted selection to breed more climate‐resilient sorghums.

## 18 MULTIPARENTAL POPULATION DISSECTS PLANT ARCHITECTURE

Genetic determinants of maize plant architecture traits influencing yield have not been well characterized. Using a newly developed maize advanced backcross‐nested association mapping population, Zhao et al. (https://doi.org/10.1002/tpg2.20179) obtained 18–88 QTL associated with plant height, ear height, and leaf angle by using three complementary mapping methods. Ten QTL hotspots wherein these three traits simultaneously coincide were identified. In addition, eight new candidate genes and four known genes were deduced in 13 major QTL regions. This study will further broaden our insights into the understanding of the genetic regulation of maize plant architecture and will help to improve maize yield and provide an invaluable resource for maize functional genomics and breeding research.

## 19 GLIRICIDIA ADAPTATION RESPONSE TO SALT STRESS

Soil salinity is one abiotic stress that threatens agriculture in more than 100 countries. Gliricidia is a multipurpose tree that originated in Central America. Carvalho da Silva et al. (https://doi.org/10.1002/tpg2.20182) report that young gliricidia plants submitted to very high salinity stress first lose all leaves to avoid death and then undergo adaptation to this abiotic stress resuming growth. Through an integrated metabolome and transcriptome analysis, the authors showed that the phenylpropanoid biosynthesis pathway plays a role in the initial response of this plant species to high salinity stress that leads later on to the adaptation response. This study is the first step on the quest to understand the genetic mechanism behind the response of gliricidia to salt stress.

## 20 A MAJOR QTL FOR SOYBEAN PARTIAL RESISTANCE TO *P. SOJAE*

Several QTL related to soybean partial resistance against *Phytophthora sojae* have been reported, but most of them have either a minor effect on the resistance level or are specific to a *P. sojae* isolate. The main limitations in the identification of QTL are linked to imprecise phenotyping assays, low genotyping resolution (mainly the SoySNP50K BeadChip), and no strong validation of the results. In this work, de Ronne et al. (https://doi.org/10.1002/tpg2.20184) report a major QTL associated with partial resistance of soybean against its most damaging pathogen, *Phytophthora sojae*, by combining a new phenotyping assay through an innovative inoculation of a mixed inoculum in hydroponics, a high‐resolution genotyping by whole‐genome resequencing of 357 soybean accessions, and a confirmation of the QTL identification by several powerful GWAS statistical models. This approach has led to the identification of a promising candidate gene whose stronger expression in lines carrying the resistance allele modulates better resistance to *P. sojae*.

## 21 LEAF RUST RESISTANCE IN WHEAT

Leaf rust is an important disease causing significant reduction in wheat production worldwide. The most efficient way to manage rust disease is to grow rust‐resistant varieties Pyramiding of minor and partial effect genes is a suitable approach to obtain durable resistance. In order to identify consensus genomic regions for leaf rust resistance, Amo and Soriano (https://doi.org/10.1002/tpg2.20185) performed a meta‐QTL analysis using 50 independent studies collecting 393 QTL. Meta‐analysis successfully reduced the genome regions to 75.

## 22 MULTITRAIT GENOMIC SELECTION AGAINST *FUSARIUM*

Fusarium head blight (FHB) caused by *Fusarium graminearium*, causes Deoxynivalenol (DON) to accumulate in the grain of affected plants. Breeding wheat varieties that resist DON accumulation is resource intensive because phenotyping multiple FHB resistance traits and measuring DON content of grain on large numbers of plots is required. Gaire et al. (https://doi.org/10.1002/tpg2.20188) discovered that multitrait genomic selection can achieve high accuracies for DON when only the percentage of fusarium damaged kernels (FDKs) is phenotyped on breeding candidates and DON and FDKs are phenotyped on the model training population. This means that instead of phenotyping multiple FHB resistance traits, breeding programs may only need to focus on phenotyping FDK and DON if they are implementing multitrait genomic selection for FHB resistance.

## 23 SALT STRESS RESPONSES IN THAI RICE CULTIVARS AND THEIR RELATIONSHIP

Salt stress affects growth performance of each rice cultivar or line differently depending on their genetic background. Habila et al. (https://doi.org/10.1002/tpg2.20189) discovered that Thai rice cultivars Jao Khao, Lai Mahk, and Luang Pratahn, had salt‐tolerance phenotypes comparable with ‘Pokkali’, the standard salt‐tolerance check, at seedlings stage. Based on phylogenetic analysis, the eight Thai rice cultivars were monophyletic and separated from Pokkali and ‘IR29’. Lai Mahk and Luang Pratahn were found closely related and had distinct genetic basis for salinity tolerance from Jao Khao. The haplotypes analysis results supported a role in salt tolerance for many genes. The identified salt‐tolerant cultivars could be used in rice breeding programs for salinity tolerance.

## 24 WHITE MOLD RESISTANCE USING A MAGIC POPULATION

White mold is one of the most important fungal diseases causing significant seed yield losses in dry bean. Genetic resistance is controlled by multiple genes with minor effects. Improved germplasm with varying levels of tolerance have been released, but no cultivars with high levels of resistance are available. Escobar et al. (https://doi.org/10.1002/tpg2.20190) developed a multiparent advanced‐generation, intercross (MAGIC) population to facilitate gene mapping and pyramiding for white mold resistance. New genomic regions associated with white mold resistance were detected while some known regions are confirmed. Pinto and great northern bean with improved resistance to white mold were identified. In addition, disease‐related candidate genes for white mold resistance were identified.

## 25 WHEAT RIBOSOMAL RNA LOCI RESOLVED

Three out of four RNA components of ribosomes are encoded by 45S ribosomal DNA (rDNA) loci, which are organized as long head‐to‐tail tandem arrays of nearly identical units spanning over several megabases of sequence. Because of this peculiar structure, the rDNA loci are the major source of gaps in genome assemblies, and expression status of particular arrays remains elusive especially in complex genomes harboring multiple loci. Tulpová et al. (https://doi.org/10.1002/tpg2.20191) coupled chromosomal genomics with optical mapping and reconstructed individual 45S rDNA arrays in bread wheat, filling in the major gaps in the reference genome and building an essential starting point for locus‐specific analyses. The engagement of individual loci in rRNA synthesis was assessed in RNA‐seq data generated for five tissues, revealing uneven and developmentally regulated activity of the loci. Results obtained in the study allowed the authors to outline a scenario of the evolutionary histories of rDNA loci in three wheat subgenomes and propose a model for the control of rRNA amount through genetic and epigenetic mechanisms.

## 26 CHROMOSOME‐SPECIFIC MARKERS IN WHEAT

The detection of wild relative introgressions in a wheat background requires robust diagnostic molecular markers. Grewal et al. (https://doi.org/10.1002/tpg2.20193) report a novel bioinformatics‐based pipeline that allows discovery of chromosome‐specific single‐nucleotide polymorphisms (SNPs) between wheat and wild relative species and their downstream use as Kompetitive allele‐specific PCR (KASP) markers for genotyping. Using this pipeline, they developed and validated a new set of KASP markers, distributed at ∼60 Mbp across the wheat chromosomes, for detecting *Amblyopyrum muticum* introgressions in a wheat background. In a set of previously developed doubled‐haploid introgression lines they found new, very small introgressions.

## 27 USING INCOMPLETE BLOCK DESIGN TO ALLOCATE LINES TO ENVIRONMENTS IMPROVES SPARSE GENOME‐BASED PREDICTION IN PLANT BREEDING

Genomic selection (GS) is a predictive methodology that has the potential to increase the speed and reduce the cost of selection. To optimize resources, sparse testing methods have been proposed by which nonoverlapping and overlapping lines are allocated randomly in locations (lines appearing in some locations but not in all). Montesinos‐Lopez et al. (https://doi.org/10.1002/tpg2.20194) propose using incomplete block designs (IBD) concept for the allocation of lines to locations in such a way that not all lines are observed in all locations. The authors compared this allocation with a random allocation of lines to locations. The authors implemented this on several crop data sets under the genomic best linear unbiased predictor model. Results provide evidence that using IBD for the allocation of lines to locations improved predictive performance compared with random allocation of lines to locations. This has the potential to be applied to large‐scale plant breeding programs.

## 28 MOLECULAR BREEDING FOR ALFALFA DROUGHT RESILIENCE

Drought threatens agricultural sustainability in many regions of the world. Singh et al. (https://doi.org/10.1002/tpg2.20195) report that DNA marker‐assisted breeding in alfalfa impacted forage biomass productivity of genetically related and genetically diverse populations during drought. When DNA marker alleles associated with forage or crown‐root biomass were identified in a given genetic background, selection for high‐biomass markers and against low‐biomass markers in genetically‐related populations frequently improved forage yield during drought. However, selection for, or against, the same marker alleles in genetically unrelated populations had varying impacts on forage biomass. Thus, confirming marker allele effects on forage productivity in genetically unrelated alfalfa populations is necessary prior to applying marker‐assisted breeding for improved drought resilience.

